# Atg7 Knockdown Augments Concanavalin A-Induced Acute Hepatitis through an ROS-Mediated p38/MAPK Pathway

**DOI:** 10.1371/journal.pone.0149754

**Published:** 2016-03-03

**Authors:** Yan Zhuang, Yi Li, Xuefeng Li, Qing Xie, Min Wu

**Affiliations:** 1 Ruijin Hospital, Shanghai Jiao Tong University School of Medicine, 197 Ruijin Road II, Shanghai 200025, China; 2 Department of Basic Biomedical Sciences, University of North Dakota School of Medicine and Health Sciences, Grand Forks, North Dakota, United States of America; French National Centre for Scientific Research, FRANCE

## Abstract

Concanavalin A (ConA), a T-cell mitogen that induces acute autoimmune hepatitis, is widely used to model pathophysiological processes of human acute autoimmune liver disease. Although autophagy has been extensively studied in the past decade, little is known about its molecular mechanism underlying the regulation of ConA-induced acute hepatitis. In this study, we used a Cre-conditional *atg7* KO mouse to investigate the effects of Atg7-associated autophagy on ConA-induced murine hepatitis. Our results demonstrated that *atg7* deficiency in mice enhanced macrophage activation and increased pro-inflammatory cytokines upon ConA stimulation. Atg7 silencing resulted in accumulation of dysfunctional mitochondria, disruption of reactive oxygen species (ROS) degradation, and increase in pro-inflammatory cytokines in Raw264.7 cells. p38/MAPK and NF-κB levels were increased upon ConA induction due to Atg7 deficiency. Blocking ROS production inhibited ConA-induced p38/IκB phosphorylation and subsequent intracellular inflammatory responses. Hence, this study demonstrated that *atg7* knockout in mice or Atg7 knockdown in cell culture augmented ConA-induced acute hepatitis and related cellular malfunction, indicating protective effects of Atg7 on regulating mitochondrial ROS via a p38/MAPK-mediated pathway. Collectively, our findings reveal that autophagy may attenuate macrophage-mediated inflammatory response to ConA and may be the potential therapeutic targets for acute liver injury.

## Introduction

Concanavalin A (ConA), the most widely used Jack bean seed lectin, is a T-cell mitogen that can induce acute autoimmune hepatitis [1,2]. ConA-induced hepatitis, which exhibits pathological changes of acute hepatic inflammation, has long been regarded as an appropriate model for studying the pathogenic mechanisms of human acute autoimmune liver disease [3,4]. It is well recognized that ConA-mediated T lymphocyte activation, followed by increased inflammatory cytokines, is the main cause of the rapid and severe inflammatory response exclusively seen in the liver. During this process, CD4^+^ T lymphocyte-mediated activation of macrophage function serves as an crucial pathogenic parameter [5]. Furthermore, a study unveiled that macrophages residing in the hepatic sinusoid were activated prior to the migration of splenic lymphocytes into the liver [6]. Kupffer cells are dedicated hepatic macrophages and functions as an essential component in liver homeostasis as well as in the acute or chronic responses to chemical compounds [7]. Activation of Kupffer cells augments the production of an array of inflammatory mediators, growth factors, and reactive oxygen species (ROS), whereas inhibition of Kupffer cell activation or depletion of Kupffer cells appears to protect against liver injury induced by immunostimulant ConA [4,8].

Autophagy is an evolutionarily conserved lysosomal pathway critical to cytoplasmic homeostasis to control the turnover of long-lived proteins and excess or dysfunctional organelles [9,10]. It occurs at basal levels, but can be induced by nutritional cues and stress signals. During the autophagic process, cellular contents including organelles are sequestered in double-membrane vesicles called autophagosomes, then the autophagosomes fuse with lysosomes where hydrolysis occurs, supplying amino acids and macromolecular precursor for cells [11,12]. Among various autophagy related genes (Atgs), Atg7, an E1-like enzyme, is essential for the autophagy conjugation system, the formation of autophagosomes and starvation-induced degradation of proteins and organelles in mammalian cells. [13,14].

To date, autophagy has been implicated in several human hepatic pathological conditions, such as hepatocellular carcinoma, HCV-associated chronic liver disease, and liver cirrhosis [15–18]. Although ConA has been demonstrated to be a potent autophagy inducer via a mitochondria-mediated pathway, the role of autophagy in ConA-induced fulminant hepatitis has been incompletely understood [19].

In this study, we utilized a Cre-conditional *atg7* KO mouse model to investigate the effect of autophagy in ConA-induced murine autoimmune hepatitis, explored the mechanisms and indicated possible ways to prevent or treat ConA-induced liver injury.

## Material and Methods

### Ethics statement

This study was carried out in strict accordance with the recommendations in the Guide for the Care and Use of Laboratory Animals of the National Institutes of Health. The protocols were approved by the Institutional Animal Care and Use Committee at the University of North Dakota, School of Medicine (Assurance Number: A3917-01). Dissections and injections were performed under anesthesia that was induced and maintained with ketamine hydrochloride (40 mg/kg) and xylazine, and all efforts were made to minimize animal suffering.

### Reagents

N-Acetyl-L-Cysteine (NAC) is clinically used as an ROS scavenger to attenuate liver injury [20]. In our study, NAC (Cat #: A7250, Sigma Aldrich, St Louis, MO) was used to inhibit ROS production. NF-κB inhibitor Sodium 4-aminosalicylate (S4073), p38 mitogen-activated protein kinase (MAPK) inhibitor SB202190 (S1077) and mTOR inhibitor rapamycin (S1039) were purchased from Selleck Company. The mitochondria toxicant Rotenone was bought from Sigma Aldrich.

### Animal experiments and ConA treatment

To investigate the physiological roles of autophagy in hepatic injury, Atg7 conditional knockout mice were employed in this study. *atg7* KO mice (*atg7*^*-/-*^) on a C57BL/6J background were graciously provided by Dr. Youwen He at Duke University. The *atg7*^*-/-*^ mice were originally generated by Masaaki Komatsu at Tokyo Metropolitan Institute of Medical Science. To conditionally delete the target gene, *atg7*^*-/-*^ mice were bred with estrogen receptor (ER) *cre* mice and were injected with 0.1 mg/kg of tamoxifen (T5648, Sigma Aldrich) daily for 6 days before experiments. Mouse genotyping for Atg7 was performed by polymerase chain reaction (PCR) analysis of tail DNA, essentially as described previously [21]. Six to eight week-old wild-type (WT) mice (C57BL/6J) were obtained from the Jackson Laboratory (Bar Harbor, ME) [22]. After acclimation for 6–7 days, mice were randomly divided into a control group and a treatment group, each containing 6 mice. Body weight was assessed at baseline.

Concanavalin A (ConA) (C2272, Sigma Aldrich) was freshly dissolved before each experiment, in pyrogen-free phosphate-buffered saline (PBS) at 2 mg/ml. ConA was injected intravenously via the tail vein of mice in the ConA model group, with equivalent volume of PBS given to the control group. For survival experiment, ConA was dosed at 40 mg/kg and was then adjusted to 20 mg/kg for modelling. Based on the results from previous reports [5] and our preliminary experiments (data not shown), we identified eighteen hours after ConA or PBS injection as an appropriate time point to assess the pathophysiology in this mouse model. Mice were executed by cervical dislocation after ketamine anesthesia (100 mg/kg body weight). Blood and liver samples were collected aseptically.

### Measurements of serum liver enzymes

Whole blood was collected by orbital sinus puncture. After centrifugation at 1, 600 × g for 10 min at room temperature, sera samples were stored at −80°C until analysis. The serum levels of alanine aminotransferase (ALT) and aspartate aminotransferase (AST) were determined by ALT activity assay kit (MAK052, Sigma Aldrich) and AST activity assay kit (MAK055, Sigma Aldrich), respectively, to evaluate the specific hepatic injury and hepatic parenchymal damage.

### Histopathology and immunohistochemically staining

Liver tissues from median and left lobes were collected, incubated in 4% paraformaldehyde (MPX00553, Thermofisher Scientific, Rockford, IL) and embedded in paraffin wax according to the traditional method. Sections were made at 4 μm thickness and stained with hematoxylin-eosin (H&E) for observation under a light microscope.

To perform immunohistochemistry staining, liver sections were prepared by heating at 70°C for 30 min. Having dewaxed in dimethylbenzene for 10 min, we dehydrated the sections in a graded series of alcohol. Antigen retrieval was then performed by microwaving the samples in citrate buffer for four cycles. Endogenous peroxidase activity was blocked by treatment with 3% H_2_O_2_ for 15 min at room temperature. Nonspecific proteins were blocked with 5% bovine serum albumin (BSA) (BP9706100, Thermofisher Scientific) for 30 min. The sections were incubated with an F4/80 (ab74383, 1:200, Abcam Company, Cambridge, MA) antibody at 4°C overnight. The sections were then washed with phosphate-buffered saline (PBS) and treated immediately with a corresponding secondary antibody at 1:500 in PBS (sc2027, Santa Cruz Biotechnology) for another 30 min. The positive reactions were finally visualized using a diaminobenzidine (DAB) kit (ab64238, Abcam) and the stained tissue was then observed under a light microscope.

### Cell viability assay

Cell viability was assessed by an MTT assay kit (M6494, Thermofisher Scientific) according to the manufacturer’s instruction. Briefly, Raw264.7 cells were seeded in a 96-well plate (21377203, Thermofisher Scientific). After ConA treatment, MTT was added to each well at a final concentration of 250 μg/ml. After 2–4 hours, the medium was removed and the cells were dissolved in DMSO (BP231100, Thermofisher Scientific) and kept overnight for complete dissolution at room temperature. Absorbance at 590nm was determined for each well using SpectraMax M5 (Molecular Devices) [23]. After subtracting the background absorbance, the A590 of the cells was divided by that of the control groups to obtain the percentage of the viable cells.

### Intracellular ROS measurement

Intracellular ROS were analyzed using hydro-dichlorofluorescein diacetate (H_2_DCF-DA) (C369, Life technologies) as probes. H_2_DCF-DA, a non-fluorescent compound, is deacetylated by viable cells to 2, 7-dichlorofluorescein (DCF) by hydrogen peroxide. Post-treatment, Raw264.7 cells were collected and incubated with 100 mM Mito-tracker Red FM/ H_2_DCF-DA (dissolved in DMSO) for 30 min at 37°C. Cells were then washed three times with PBS and the intracellular accumulation of fluorescent DCF-DA was measured by LSM 510 Meta confocal laser scanning microscope (CLSM) (Carl Zeiss MicroImaging, Thornwood, NY). The results were expressed as the intensity of DCF fluorescence.

### Mitochondrial membrane potential Assay and Superoxide Dismutase (SOD) Enzyme Activity Assay

The mitochondrial membrane potential was measured by JC-1 staining (T3168, Life technologies) bearing a delocalized positive charge that can enter the mitochondrial matrix due to the negative charge established by the intact mitochondrial membrane potential. After incubation with JC-1 dye, fluorescence at 485 nm was measured by a fluorometer (BioTek, Winooski, VT). The level of SOD in Raw264.7 cells were analyzed by commercially available SOD kits (19160, Sigma Aldrich) according to the manufacturer’s instruction.

### ELISA

The levels of TNF-α, IL-1β, IFN-γ and IL-6 in murine plasma or Raw264.7 cell suspensions were analyzed by ELISA using commercially available kits (eBioscience, San Diego, CA) according to the manufacturer’s instruction.

### Cell transfection and confocal microscopy

Raw264.7 cells were respectively transfected with Atg7 siRNA (S20651, Invitrogen, Grand Island, NY) or GFP-LC3 plasmid, using LipofectAmine 2000 reagent (11668019, Invitrogen) in serum-free RPMI 1640 medium (MT15040CV, Thermofisher Scientific) per the manufacturer’s instructions. For immunostaining, Raw264.7 cells were grown in glass-bottomed dishes (MatTek, Ashland, MA) and fixed in 4% paraformaldehyde, permeabilized with 0.2% Triton X-100 in PBS and incubated with blocking buffer for 30 min, then cell nuclei were stained with 2-(4-amidinophenyl)-6-indolecarbamidine dihydrochloride (DAPI) (1:1000 dilution of stock in PBS) for 5 min [24]. The cells were incubated with a p65 antibody (sc7151, Santa Cruz Biotechnology) at 1:500 dilution in blocking buffer overnight and washed three times with wash buffer. Images were captured after incubation with an appropriate secondary antibody containing FITC using a 510 Meta confocal microscope (Carl Zeiss MicroImaging), and processed using the software provided by the manufacturer [25].

### Western blotting

Protein concentrations were determined by the BCA Protein Assay Kit (Bio-Rad, Hercules, CA) [26]. Total proteins were extracted from the tissues and cells using a RIPA lysis buffer method and stored at −80°C until western blotting analysis. After boiled at 100°C for 5 min, an equal amount of protein samples was loaded for sodium dodecyl sulfate polyacrylamide gel for electrophoresis (SDS-PAGE), and then transferred to nitrocellulose membranes, blocked with 5% skim milk for 1 h at room temperature, and then incubated with primary antibodies at 4°C overnight. After the samples were incubated with a secondary antibody for 120 min at room temperature, the signals were detected with the chemiluminescence (34096, SuperSignal West Femto Maximum Sensitivity Substrate, Thermofisher Scientific).

### Electrophoretic Mobility Shift Assay

Nuclear extracts were isolated following the instruction of NE-PER Nuclear and Cytoplasmic Extraction Reagents Kit (78833, Pierce-Thermofisher Scientific). After preparation of nuclear extracts, the biotin end-labeled DNA was subjected to gel electrophoresis on a native polyacrylamide gel and transferred to a nylon membrane, then detected using a LightShift Chemiluminescent EMSA kit (20148, Pierce-Thermofisher Scientific) as previously described [27]. The NF-κB p65 probe used for this experiment was purchased by Viagene Biotech.

### Statistical analysis

All experiments were performed in triplicate and repeated at least three times. Data were presented as ratios or percentage changes compared with mean+SD from the three independent experiments. Parametric data were statistically analyzed by the Student’s *t* test or one-way ANOVA followed by post hoc tests when appropriate. Differences in Non-parametric data were evaluated by the Mann-Whitney *U* test. Survival curves were analyzed using Kaplan-Meier test. *p*<0.05 was used to indicate a statistically significant difference. The calculations were performed with the SPSS software, version 11.0 (SPSS, Inc., Chicago, IL).

## Results

### *atg7* deficiency augmented ConA-induced murine acute hepatic injury

**Fig 1:**
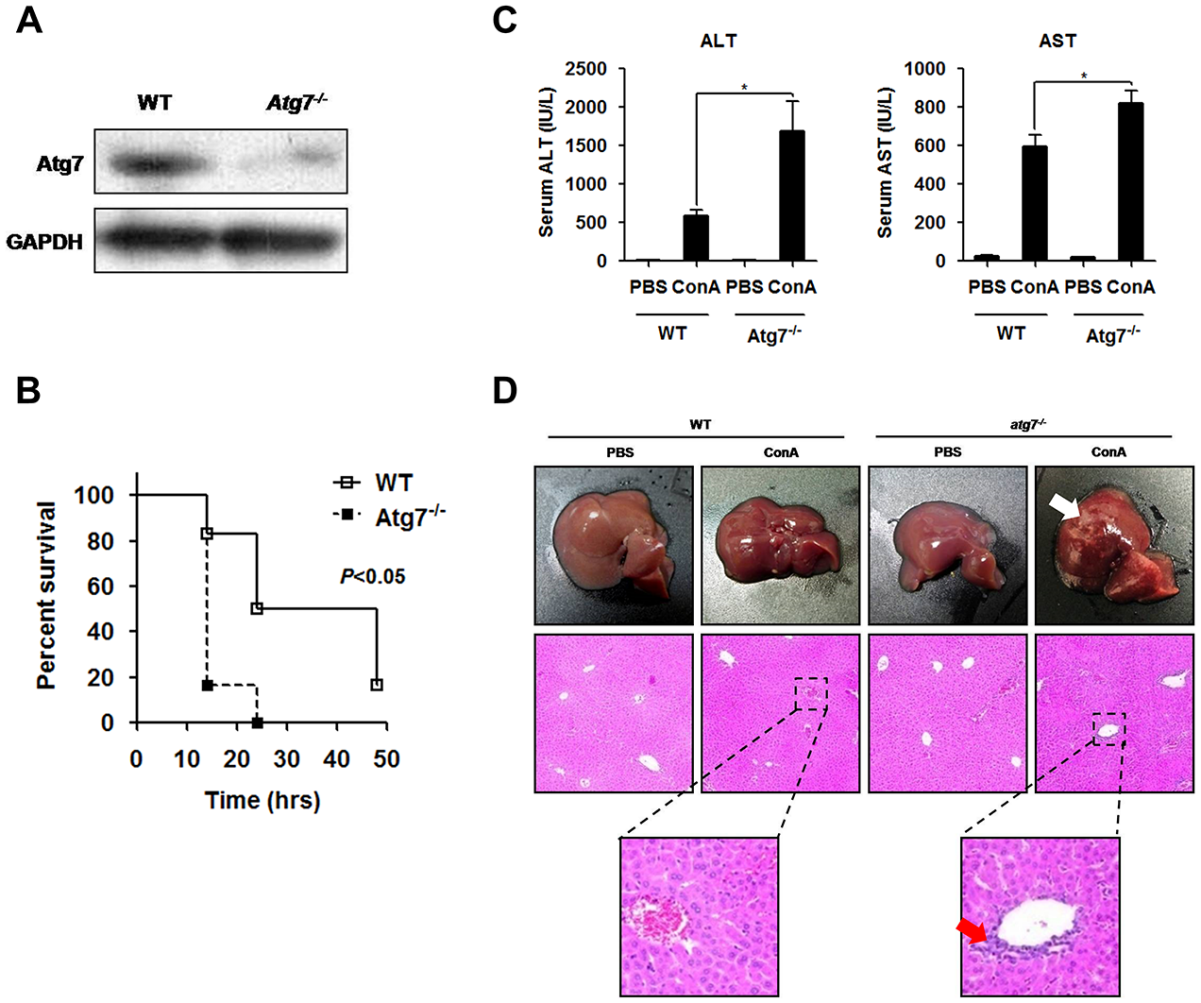
*atg7* deficiency augmented ConA-induced murine acute hepatic injury. **A.** Western blotting of Atg7 protein expression in the liver of *atg7*^*-/-*^ mice and the littermate controls. **B.** Survival curve of the two groups following ConA injection. Mice were challenged with 40 mg/kg ConA and then observed every 2 h until 48 h. **C.** Serum ALT and AST levels after ConA injection. **D.** Histopathological staining of liver sections from *atg7*^*-/-*^ mice and the littermate controls. Representative results were from 3 independent experiments. Data are presented as mean±SD (n = 6), * *p*<0.05 vs. littermate controls.

To examine the role of *atg7*-depleted autophagy in the pathogenesis of ConA-induced autoimmune hepatitis, tamoxifen-induced Cre-mediated *atg7* mutant *(atg7*^*-/-*^) mice and its littermate controls were employed in this study. Atg7 protein was expressed at background levels in the liver of *atg7*^*-/-*^ mice (Fig 1A). When mice were challenged with 40 mg/kg of ConA, a lethal dose reported by Tiegs G [28], a robust statistically significant difference in survival rates was observed between the two groups. *atg7*^*-/-*^ mice manifested worse outcomes compared with control mice (Fig 1B). Based on the results from previous study [5] and preliminary experiments (data not shown), we utilized ConA at a lower dose of 20 mg/kg to construct acute hepatitis model in our study. Serum aminotransferase levels were markedly elevated in *atg7*^*-/-*^ mice compared to the littermate controls (Fig 1C). Histopathological changes were consistent with the liver enzyme results. Though hepatic injury, including hepatocytic swelling, degeneration vacuolization and neutrophil infiltration, was apparently observed in both groups, liver damage was more serious in *atg7*^*-/-*^ mice (Fig 1D). These results delineated that depletion of *atg7* increased susceptibility to ConA hepatitis in mouse models.

### *atg7* deficiency increased the production of pro-inflammatory cytokines following ConA injection

**Fig 2:**
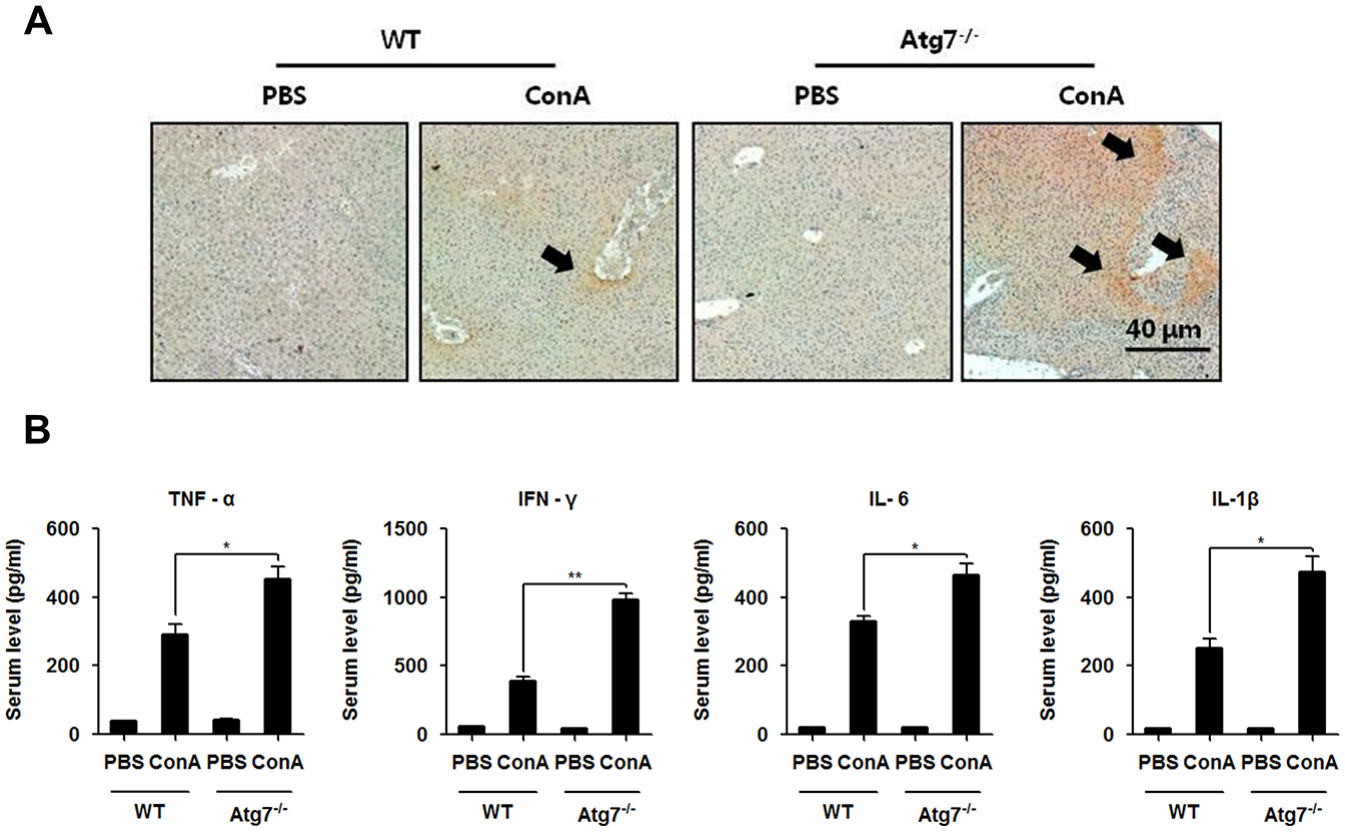
*atg7* deficiency increased the production of pro-inflammatory cytokines following ConA injection. **A.** The immunochemistry micrographs stained by F4/80 mAb of liver tissues from *atg7*^*-/-*^ mice and the littermate controls at 18 h following ConA injection (Arrows showing inflammatory regions). **B.** ELISA analysis of serum TNF-α, IFN-γ, IL-6 and IL-1β 18 h after ConA injection. Representative results were from 3 independent experiments. Data are presented as mean±SD (n = 6), * *p*<0.05 and ** *p*<0.01 vs. littermate controls.

Pro-inflammatory cytokines are essential in the pathogenesis of ConA hepatitis. To investigate whether and how resident hepatic macrophages, Kupffer cells, were activated in this setting, mouse macrophage-specific marker F4/80 mAb was used to visualize the phenotypic characterization of mature macrophages in the liver. Indeed, we observed an increase in the F4/80 positive cells in *atg7*^*-/-*^ mice than the littermate controls (Fig 2A). To further explore the mechanism of *atg7* deficiency in intra-hepatic inflammation, serum pro-inflammatory mediators were assayed. Lacking of Atg7 in mice led to an increased production of circulatory TNF-α, IFN-γ, IL-6 and IL-1β as measured by ELISA (Fig 2B). Thus, it is suggested that worsened hepatic injury upon *atg7* deficiency in ConA hepatitis may be resulted from over activation of macrophages and excessive production of pro-inflammatory cytokines.

### Inhibition of autophagy via Atg7 silencing resulted in accumulation of dysfunctional mitochondria and abolished ROS degradation

To validate the observations derived from animal experiments, we used immortalized murine macrophages, Raw264.7 cells, as an *in vitro* model to investigate the role of Atg7-related autophagy in ConA-induced macrophage activation. ConA has been shown to be a potent autophagic inducer [29–32]. In preliminary experiments, we confirmed that autophagy was induced in Raw264.7 cells when incubated in the complete media supplemented with ConA and was inhibited when Atg7 was knocked down by siRNA silencing (S1A Fig and Fig 1B).

**Fig 3:**
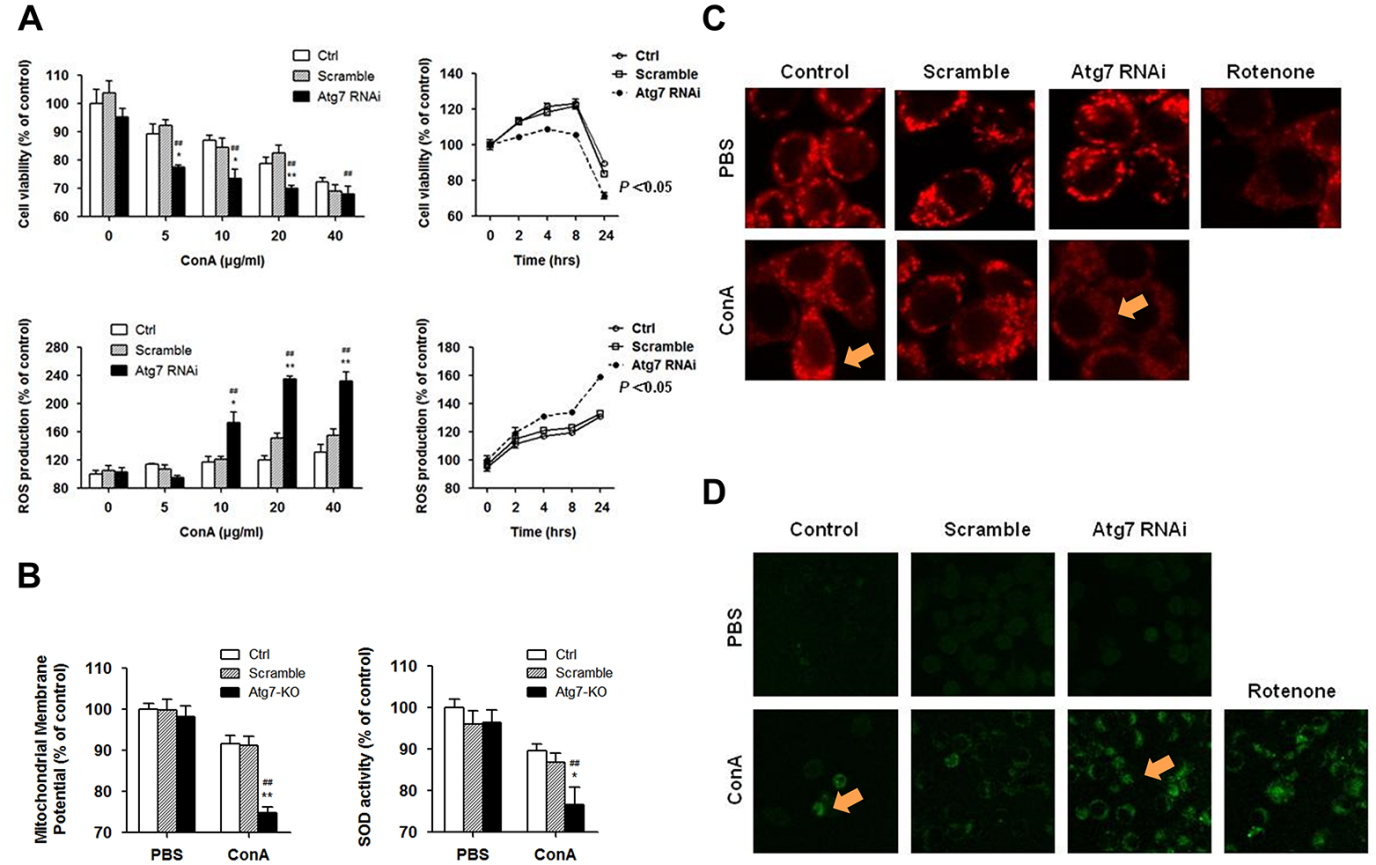
Atg7 silencing resulted in accumulation of dysfunctional mitochondria and abolished ROS degradation in Raw264.7 cells. **A.** Cell viability measured by an MTT assay and ROS production by mean fluorescence intensity of H_2_DCF-DA at different concentrations and time points for ConA exposure in Raw264.7 cells lacking Atg7. **B.** Measurement of mitochondria membrane potential and SOD activity after ConA stimulation. Raw264.7 cells were transfected with Atg7 siRNA or control siRNA and treated with 10 μg/ml ConA for 24 h. Representative results were from 3 independent experiments. Data are presented as mean±SD; * *p*<0.05 and ** *p*<0.01 vs. controls, # *p*<0.05 and ## *p*<0.01 vs. pre-treatments. **C and D.** Fluorescence microscopy of mitochondrial membrane by Mito-tracker Red FM (red) and ROS production by H_2_DCF-DA (green) after ConA stimulation (Arrows showing fluorescence staining regions). Raw264.7 cells were transfected with Atg7 siRNA or control siRNA and treated with 10 μg/ml ConA for 4 h. Magnifications: 1000X. Representative results were from 3 independent experiments.

Raw264.7 cells were then stimulated with ConA at different concentrations and time points. We found that ConA increased intra-cellular ROS accumulation and decreased cell viability in a concentration-dependent manner, whereas these changes were more significant upon Atg7 depletion. For the time-dependent tests, cell viability slightly increased and peaked at earlier times of ConA exposure and decreased to a lower level 24 h post-treatment in Atg7-silenced cells than in controls. By contrast, intra-cellular ROS levels rose over time and reached a higher level in Atg7-silenced cells. Loss of autophagy by *atg7* silencing exhibited significant decreases in cell viability and increases ROS production 24 h post-treatment (Fig 3A).

We then evaluated whether these ConA-induced alterations were mitochondria-associated. ConA 10 μg/ml for 24 h was chosen for a detailed mechanistic study. Mitochondrial membrane potential and SOD activity were detected pre- and post-ConA treatment, respectively. Exposure to ConA decreased mitochondrial membrane potential and SOD activity, which was further reduced in Atg7-silenced Raw264.7 cells (Fig 3B).

To further validate our observation, fluorescence microscopy was used to visualize the phenotypic characterization of cultured cells lacking Atg7. Exposure to ConA greatly impaired the mitochondrial membrane as reflected by reduction in the red fluorescent intensity of Mito-tracker Red FM. The alteration was more significant in Atg7-silenced cells when compared to controls (Fig 3C). The contents of ROS in Raw264.7 cells were similarly determined with H_2_DCF-DA. The green fluorescence started to increase upon ConA exposure and appeared to be stronger in Atg7-silenced cells when compared to control cells (Fig 3D). These findings confirmed that ConA-induced mitochondrial dysfunction and viscous ROS accumulation in Raw264.7 cells, which were worsened by Atg7 knockdown.

### Atg7 knockdown extravagantly activated p38/MAPK and NF-κB signaling in Raw264.7 macrophages

**Fig 4:**
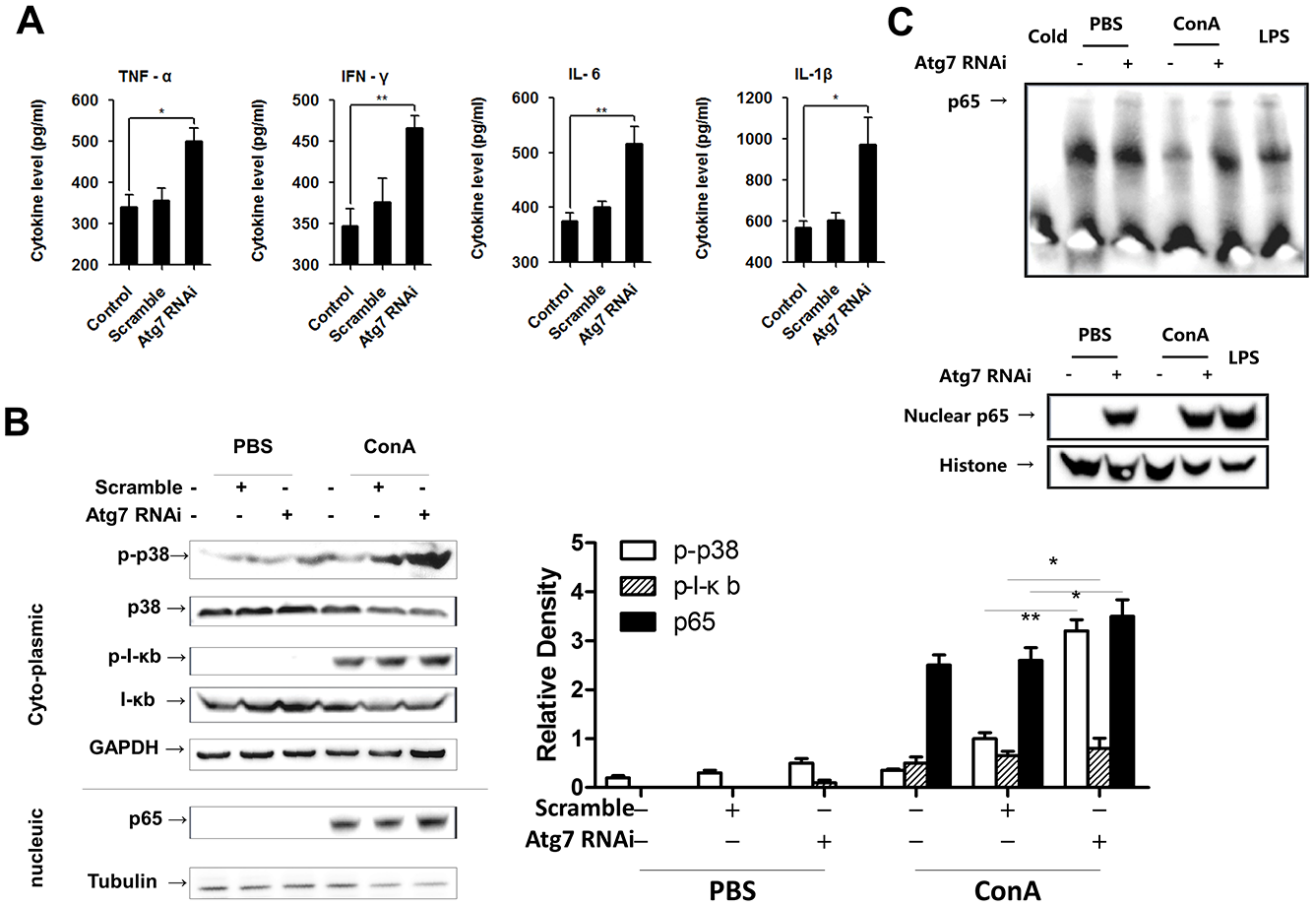
Atg7 knockdown extravagantly activated p38 and NF-κB signaling in Raw264.7 macrophages. **A.** ELISA analysis of pro-inflammatory cytokines in the supernatant of cell cultures upon ConA challenge. **B.** Western blotting analysis of p-p38, p-IκB and nuclear p65 protein expression in cultured cells upon ConA challenge. Differences in p38 and IκB phosphorylation were quantitated using densitometry. * *p*<0.05 and ** *p*<0.01 vs. controls. **C.** EMSA results of NF-κB p65 activation and translocation upon ConA challenge. Raw264.7 cells were transfected with Atg7 siRNA or control siRNA and treated with 10 μg/ml ConA for 2 h. Representative results were from 3 independent experiments. Data are presented as mean±SD; * *p*<0.05 and ** *p*<0.01 vs. controls.

Based on the finding that an Atg7-dependent process was specifically involved in the ROS-associated cell function and death in macrophages, we thus switched our focus onto the interface between autophagy and ConA-induced inflammatory signaling pathway in this setting. We first measured the levels of pro-inflammatory cytokines in the supernatant of cell cultures. As expected, TNF-α, IFN-γ, IL-6 and IL-1β were all significantly elevated in Raw264.7 cells lacking Atg7 (Fig 4A).

It is recognized that the p38/NF-κB signaling pathway plays important roles in regulating hepatic inflammatory responses [20,33,34]. In our initial experiments, the kinetics of p-p38/p38, rather than p-JNK/JNK and p-ERK/ERK, was found to be consistent with inflammatory changes in Raw264.7 cells after ConA challenging. By immunofluorescence staining, we confirmed the translocation of NF-κB p65 into the nucleus of Raw264.7 cells, irrespective of Atg7 (S2 Fig). When the expression levels of cytosolic p-p38/p-IκB and nucleic NF-κB p65 were measured by western blotting, we found that p-p38 and p-IκB were surprisingly up-regulated by ConA stimulation. These changes were more significant in Atg7-silenced cells (Fig 4B). We next performed EMSA to examine the conjugation of NF-κB p65 with the promoter gene in the nucleus. In line with previous results, EMSA also exhibited an augmented NF-κB p65 activation, nuclear translocation and combination with the target gene in immortalized macrophages lacking Atg7 (Fig 4C).

### Blocking ROS production reduced ConA-induced p38/IκB phosphorylation and subsequent pro-inflammatory responses in Atg7-silenced macrophages

**Fig 5:**
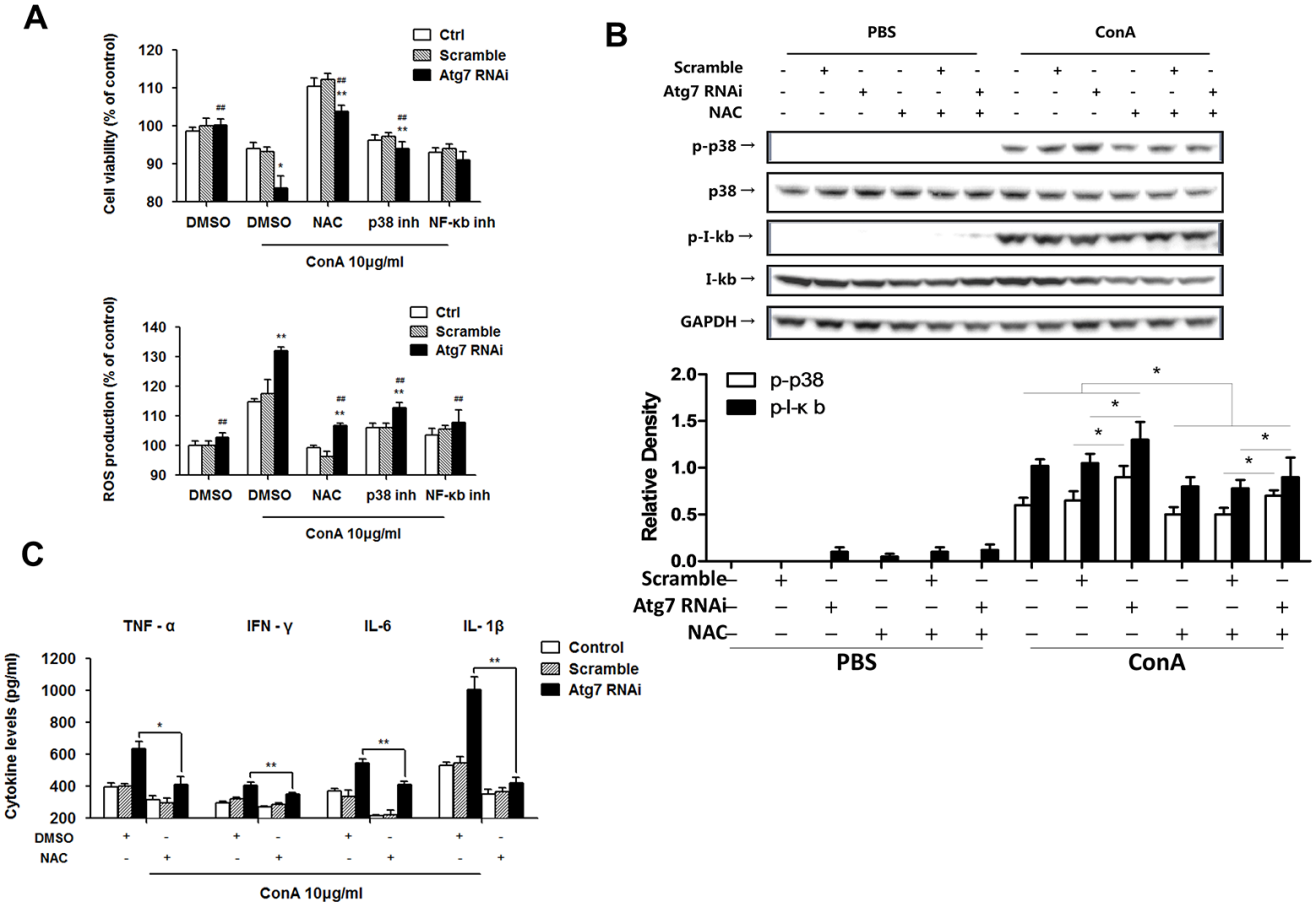
Blocking ROS reduced ConA-induced p38/IκB phosphorylation and subsequent pro-inflammatory responses in Atg7-silenced macrophages. **A.** Raw264.7 cells were pretreated with NAC (1 mM), SB202190 (10 μM) and Sodium 4-aminosalicylate (10 μM), respectively, for 30 min prior to 10 μg/ml ConA treatment for 24 h. Cell viability was measured by an MTT assay and ROS production detected by H_2_DCF-DA. **B.** Western blotting on p-p38 and p-IκB phosphorylation levels in cultured cells. Densitometry of quantification of p-p38 and p-IκB phosphorylation. * *p*<0.05 and ** *p*<0.01 vs. controls, # *p*<0.05 vs. non NAC-treatments. **C.** ELISA of TNF-α, IFN-γ, IL-6 and IL-1β levels in the supernatant of cultured cells. Raw264.7 cells were transfected with Atg7 siRNA or scrambles siRNA, pretreated with 1 mM NAC for 30 min and then treated with 10 μg/ml ConA for 24 h. Representative results were from 3 independent experiments. Data are presented as mean±SD; * *p*<0.05 and ** *p*<0.01 vs. control, # *p*<0.05 and ## *p*<0.01 vs. ConA alone.

To verify the effect of ROS in mediating p38/NF-κB pathway in macrophages, we employed chemical inhibitors (ROS inhibitor NAC, p38 inhibitor SB202190 and NF-κB inhibitor Sodium 4-aminosalicylate). Cell viability and ROS production upon ConA stimulation were measured in Raw264.7 cells lacking Atg7. Although siRNA-mediated knockdown of Atg7 led to an increase in ROS production and decrease in cell viability, NAC exhibited the strongest inhibition in ROS among the three differently pre-treated cell groups (Fig 5A). Western blotting shows that addition of NAC reversed over-phosphorylation of p38 and IκB (Fig 5B). The significant alterations in phosphorylation of p38 and IκB were quantitated using densitometry (Fig 5C). Furthermore, NAC also reduced subsequent overproduction of pro-inflammatory cytokines, including TNF-α, IFN-γ, IL-6 and IL-1β upon ConA stimulation in Raw264.7 cells (Fig 5D).

**Fig 6:**
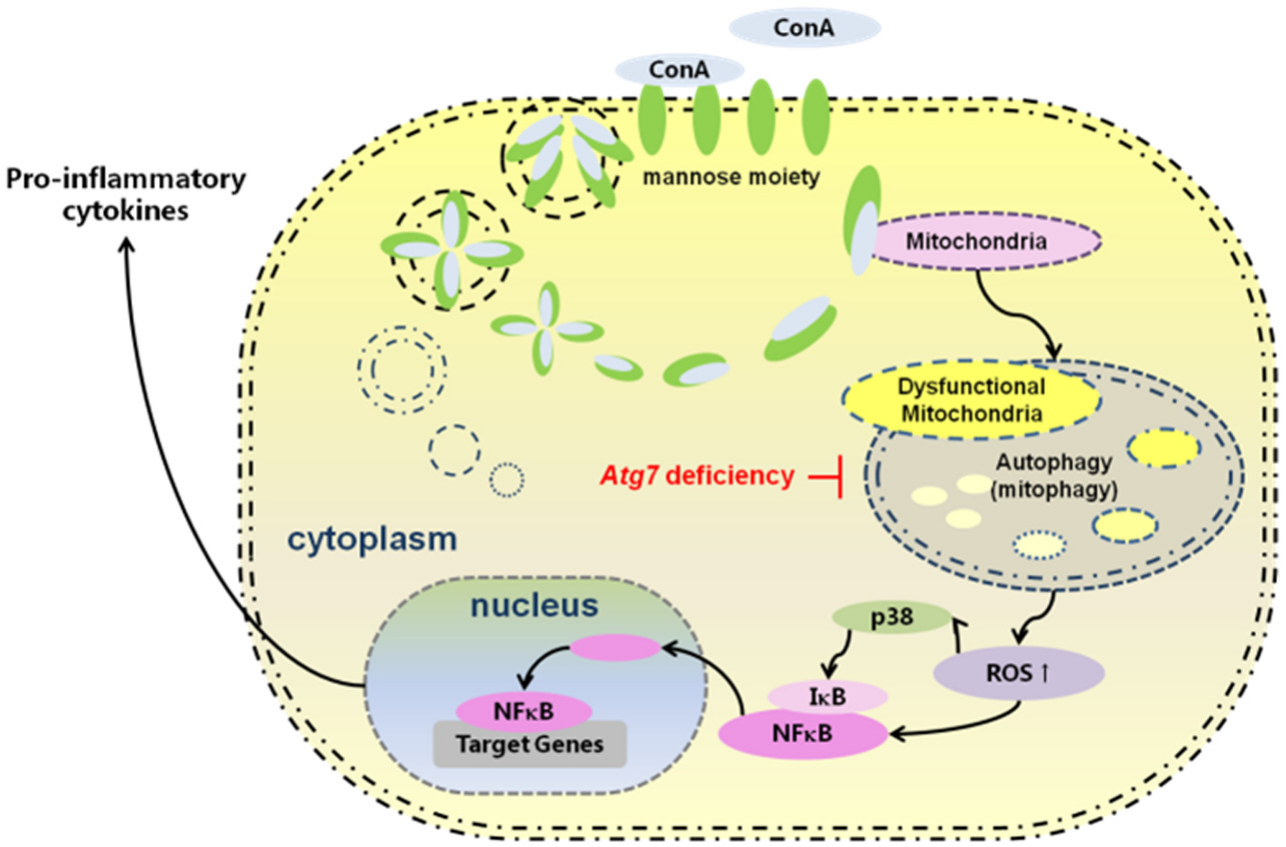
Putative mechanism in which Atg7 knockdown augments ConA-induced hepatic injury through an ROS-mediated p38/MAPK pathway in intra-hepatic macrophages.

These findings indicate that ConA-induced ROS are associated with p38/IκB phosphorylation in macrophages, which is responsible for pro-inflammatory responses. We put forward to a mechanistic model by which Atg7 effects in the center stage in controlling proinflammatory response in liver hepatitis induced by ConA (Fig 6).

## Discussion

The current understanding of mammalian autophagy in the liver stems mainly from studies of cancer [29,32] and ischemia/reperfusion injury [35–37], whereas the autophagic mechanism in the pathogenesis of acute autoimmune hepatitis has barely been investigated. Although the regulatory effect of autophagy in liver injury remains elusive, a growing amount of researches have demonstrated that autophagy plays a protective role in hepatocytes under stress conditions. Complete activation of autophagic process attenuates liver injury and improves survival in septic mice [38,39]. In this study, using a tamoxifen-induced conditional *atg7*^*-/-*^ mice, we investigated the role of Atg7 in the pathogenesis of ConA-induced autoimmune hepatitis. Thus, we uncovered that autophagy disruption by *atg7* depletion increased mortality and enhanced liver damage in mice models.

It is well established that ConA-induced liver injury is initiated by specific activation of CD4^+^ lymphocytes, which is followed by activation of macrophage function and release of inflammatory cytokines, resulting in necrosis and apoptosis of hepatocytes [5,40,41]. However, a recent study unveils that Kupffer cell plays an important role in T cell activation-induced liver injury by impacting TNF-α production. Liver damage was restricted and TNF-α levels were unaltered when Kupffer cells were depleted by clodronate liposome [8]. In our study, the intensity of immuno-histochemistry staining of macrophage-specific marker F4/80 in liver samples were consistent with the severity of liver injury, indicating that macrophages may serve as a critical inflammatory and immune responder in ConA-induced hepatitis, whose cooperative signaling is indispensable for this pathogenic process. Meanwhile, *atg7* deficiency promoted activation of macrophages, which is likely to be responsible for exacerbated systematic inflammatory response and liver injury in *atg7*^*-/-*^ mice. Based on the *in vivo* results, we hypothesize that autophagy is, at least partially, involved in the pathophysiology of ConA hepatitis in Kupffer cells.

In order to further investigate the regulatory role of autophagy in Kupffer cells in ConA-induced autoimmune hepatitis, immortalized macrophage cell line, Raw264.7 cells were used. We found that ConA treatment increased intra-cellular ROS generation and decreased cell viability in a time- and concentration-dependent manner in cultured cells. Spectrophotometric analysis on mitochondrial membrane potential and SOD activity showed an exaggerated mitochondrial damage with Atg7 knockdown. Similar results about ROS quantity and mitochondrial morphological alteration were obtained using fluorescence microscopy analysis.

It has been previously reported that aberrant mitochondrial function and disrupted ROS clearance activate the p38 MAPK pathway [42]. In addition, the p38 MAPK pathway acts as a key regulator in immune and inflammatory responses in ROS-mediated autophagy [43] and apoptosis [34]. Here, we confirmed that inhibition of autophagy augmented ConA-induced inflammatory response via a ROS-mediated pathway, demonstrated by up-regulated activation of p38/IκB, which in turn led to global inflammatory responses and cell death in cultured cells. Given that ConA-induced hepatitis can be ameliorated by antioxidants [44–46], we used ROS, p38 and NF-κB inhibitors to investigate the importance of ROS-mediated signaling in macrophages upon ConA challenging. The administration of NAC, a ROS inhibitor, appeared to robustly correct defects in ROS degradation, leading to improved cell viability, downregulated p38/IκB phosphorylation, and, ultimately, reduced pro-inflammatory cytokine secretion. We thus concluded that enhanced ConA signaling might have resulted from the accumulation of dysfunctional mitochondria and elevated ROS levels, which can be blocked by NAC. Together, these results suggest that Atg7-associated autophagy indeed plays a protective role in response to ConA stimulation by interfering with the p38 MAPK pathway to control the overzealous pro-inflammatory response in the liver.

Intriguingly, although p38 and NF-κB inhibitors also blocked ROS production, such inhibition could not confer capacity to suppress ROS in Atg7-silenced cells to the levels found in control counterparts, indicating that significant part of dysfunctional mitochondria and altered ROS generation are constitutively degraded in the lysosome via an autophagic pathway. In addition, NAC did not substantially inhibit the phosphorylation of p38/ IκB, implying that the activation of the ROS-mediated p38 MAPK pathway was significantly but not exclusively involved in ConA-induced inflammatory response in macrophages. ROS is a group of highly reactive molecular forms of oxygen that are continuously produced as a byproduct of the mitochondrial respiratory chain [47–49]. It is usually maintained at tolerable levels in normal conditions. Imbalance due to either increased ROS production or decreased ROS degradation can cause excessive ROS accumulation, which evokes damage to cell structures and compromising cellular function [50–53]. ROS are recognized as the crossroads of mitochondria, inflammation and autophagy signaling pathways [53]. Autophagy is one of the major intra-cellular pathways by which dysfunctional mitochondria are eliminated [54]. Inhibition of autophagy promoted mitochondrial ROS accumulation, which further aggravated mitochondrial damage. These changes represent a vicious cycle that accelerated the process of cell death and inflammatory pathway activation.

Hence our study demonstrates that *atg7* deficiency augmented ConA-induced murine acute hepatitis, indicating a protective effect of Atg7 on regulating mitochondrial ROS degradation via a p38 MAPK pathway. These findings may indicate novel therapeutic targets for fulminant hepatitis. Given that a variety of mechanisms may be involved in the pathogenesis of autoimmune hepatitis, further studies are required to elucidate the mechanisms of *atg7* deficiency relating to T cells upon ConA stimulation.

## Supporting Information

S1 Fig Atg7 knockdown in Raw264.7 cells impaired autophagy. **A.** Western blotting analysis of Atg7, p63 and LC3 protein expression in Raw264.7 cells after ConA (10 μg/ml) treatment for 2 h. Disrupted consumption of p62 and interrupted conversion of LC3-II to LC3-I were observed in Atg7 siRNA transfected cells. **B.** Raw264.7 cells transfected with GFP-LC3 plasmid were treated with ConA (10 μg/ml) for 4 h (Arrows showing GFP-LC3 punctate regions). Rapamycin (50 nM) treatment for 24 h was used as positive control. GFP-LC3 punctate, the marker of autophagosome formation, was scarce to none in Atg7-silenced cells. Magnifications 1000X. Representative results were from 3 independent experiments. Data are presented as mean+SD; * *p*<0.05 vs. controls.
(TIF)

S2 Fig Translocation of NF-κB p65 into the nucleus of Raw264.7 cells. Immunofluorescence staining shows no significant difference in translocation of NF-κB p65 between Atg7-silenced cells or controls.
(TIF)
