# Supplementary figures and images for: Atg7 Knockdown Augments Concanavalin A-Induced Acute Hepatitis through an ROS-Mediated p38/MAPK Pathway
Source: PLoS One. 2016 Mar 3;11(3):e0149754. doi: 10.1371/journal.pone.0149754 (PMC4777387; doi:10.1371/journal.pone.0149754)

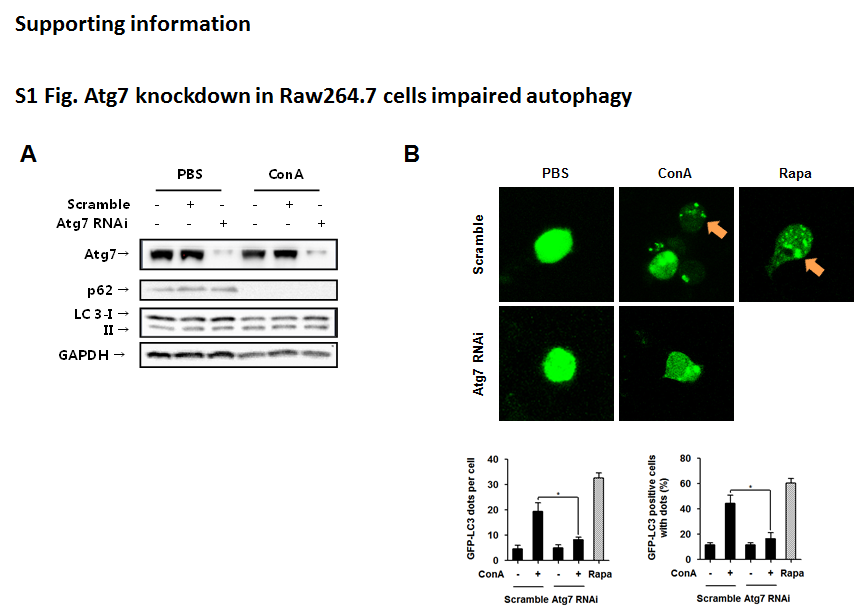

Supplement: S1 Fig — A. Western blotting analysis of Atg7, p63 and LC3 protein expression in Raw264.7 cells after ConA (10 μg/ml) treatment for 2 h. Disrupted consumption of p62 and interrupted conversion of LC3-II to LC3-I were observed in Atg7 siRNA transfected cells. B. Raw264.7 cells transfected with GFP-LC3 plasmid were treated with ConA (10 μg/ml) for 4 h (Arrows showing GFP-LC3 punctate regions). Rapamycin (50 nM) treatment for 24 h was used as positive control. GFP-LC3 punctate, the marker of autophagosome formation, was scarce to none in Atg7-silenced cells. Magnifications 1000X. Representative results were from 3 independent experiments. Data are presented as mean+SD; * p<0.05 vs. controls. (TIF) [file pone.0149754.s001.tif]

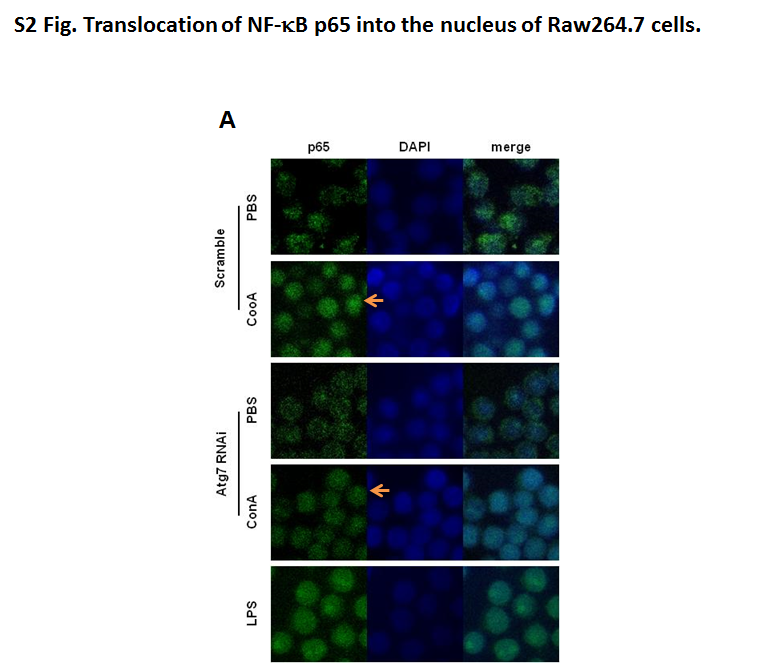

Supplement: S2 Fig — Immunofluorescence staining shows no significant difference in translocation of NF-κB p65 between Atg7-silenced cells or controls. (TIF) [file pone.0149754.s002.tif]
